# Prevalence of *Trypanosoma cruzi* Infection among People Aged 15 to 89 Years Inhabiting the Department of *Casanare* (Colombia)

**DOI:** 10.1371/journal.pntd.0002113

**Published:** 2013-03-07

**Authors:** Fredy Roberto Salazar Gutierrez, Martha Liliana Trujillo Güiza, Magally del Carmen Escobar Martínez

**Affiliations:** Biomedical Sciences Research Group, School of Medicine, Universidad Antonio Nariño, Bogotá, Colombia; US Food and Drug Administration, United States of America

## Abstract

The purpose of this study was to calculate the seroprevalence of *Trypanosoma cruzi* infection in a sample of inhabitants from a region considered to be at high risk of natural transmission of Chagas disease in Colombia. A cross-sectional study was conducted in subjects from 5 municipalities, recruited in urban and rural locations, distributed by gender according to the demographic information available. Socio-demographic information, history of potential exposure to insect vectors, blood donating, as well as symptoms suggesting cardiac disease were collected using a questionnaire. After giving written informed consent, blood specimens were obtained from 486 people to determine the serologic evidence of past exposure to *T. cruzi*. Infection was diagnosed when two different tests (ELISA and IHA) were positive. The seroprevalence of antibodies against *T. cruzi* was 16.91% considering an estimated population of 44,355 aged between 15 and 89 years (95%IC: 13.72 to 20.01). The factors significantly associated with the infection were: 1- Housing materials like vegetable material, adobe or unfinished brick walls; 2- The fact of having previous tests for Chagas disease (regardless of the result). Of note, the mean ages among infected and not infected participants were significantly different (49.19 *vs.* 41.66, p≤0.0001). Among the studied municipalities, the one with the highest frequency of *T. cruzi* infection was Nunchia, with 31.15% of the surveyed subjects. Therefore it may be concluded that *T. cruzi* infection is highly prevalent in the north region of *Casanare*, in Colombia.

## Author Summary

Chagas disease is caused by the chronic infection with the parasite *Trypanosoma cruzi* and is transmitted to human beings by bloodsucking insects of the *Triatoma* family. This condition is endemic in Central and South America, and is acquiring relevance in the rest of the world since it can also be transmitted by transfusions or organ transplantation from asymptomatic, infected donors. Detecting infected persons is one of the most important points in public health, mostly because it allows the identification of infected individuals at earlier stages of the disease, where adequate medical surveillance will permit secondary prevention measures to be given to these patients and thus avoidance of disease complications such as cardiac arrhythmia and heart failure. In this study we describe the prevalence of infection in a region of Colombia that was previously considered at risk for the infection because of the presence of infected insect vectors living in housings.

## Introduction

Chagas disease is caused by the protozoan hemoflagellated *Trypanosoma cruzi*. The infection is transmitted to humans or many other vertebrates by insects of the *Triatoma* family, usually through the deposit of infecting forms of the parasite on the stool, near mucosae or minimal lesions in the skin [1]. Recently, transmissions through contaminated food [2] or by tissue transplantations [3] as well as congenital infection [4] have called the attention of health authorities even in countries where the natural transmission does not occur [5].

According to estimates from the Pan American health organization (PAHO), among 100 million people that live in 21 endemic countries, 90 million are at risk of contracting the infection and 10 million are already infected [6]. This condition is still considered the fourth leading cause of mortality in Latin America, causing about 10,000 deaths each year [6]. In Colombia, it has been estimated that about 1 million people are infected and 3 million are at risk of acquiring the infection [7], [8]. Of note, the lack of accurate markers of disease progression makes difficult to predict who of them will suffer from cardiomyopathy. Despite the multiple efforts that have been made to eradicate the insect vectors from rural housings [8], a number of factors may be acting to favor the persistence of some endemic foci of this zoonosis in Latin American countries. As a consequence, Chagas disease is a preventable condition that still affects the poorest populations living in rural areas.

The onset of cardiac or digestive disease may be settled after several years of the infection event, and it is manifested in about 30–40% of individuals as a progressive dilated chronic cardiomyopathy (CCC). The digestive forms of the disease are less frequently seen, and they may rarely coexist with CCC. Therapeutic approaches remained unchanged for more than 20 years and their utility to halt cardiac derangements induced in Chagas disease patients or even their effectiveness to protect asymptomatic patients from cardiac manifestations of the disease remains uncertain [9].

One important aspect of *T. cruzi* infection which may contribute to its spreading is the absence of clinical symptoms over decades. In fact, immigrants from Latin-America that act as tissue donors may have contributing to the transmission of the disease in countries where insect vectors are not found [10]. Although the screening for Chagas disease is currently mandatory in blood banks in endemic countries, data obtained from not endemic countries suggest that the efficiency of transmission of the infection when a transfusion is made from infected donors is between 12 to 48% [11]. Additionally, infected asymptomatic women may also transmit the disease to their children during pregnancy or during the birth. It is estimated that 1–12% of newborns of infected mothers could be infected [4]. A recent study found a prevalence of infection of 4% among pregnant women in *Casanare* [12]. Hence, the identification of infected people is of most importance for public health authorities, in order to know the real burden of the disease, as well as to establish measures towards secondary prevention for transfusion-borne and congenital Chagas disease.

Regional initiatives directed toward the eradication of the insect vectors have been well succeeded in some countries [13], [14]. Among these initiatives, the Andean regional program for control of Chagas disease has ended in 2006 [15], with a slight impact on the control of natural transmission of Chagas disease in some regions of Colombia. In this country, most of the available epidemiological data on the prevalence of *T. cruzi* infection in humans is from blood banks, showing prevalences of the infection between 3 and 6% [16]–[21]. Though, leaving to these centers the responsibility to detect positive cases may not be helpful if we want to know the real burden of the disease and if secondary measures for prevention are planning to be settled. Studies concerning the performance of the serological tests used in the screening of donors in blood banks from all the country indirectly found in 2002 and 2003 that Casanare was the region with the highest presence of confirmed infection (12.6% and 5.04% respectively) among donors in the blood bank of the municipality of Yopal. These studies used the same mixtures of recombinant antigens of ELISA sets that were used in our study, to confirm the infection state of the individuals [20], [21].

The frequency of Chagas disease and *T. cruzi* infection in people remains largely unknown in Colombia, even when previous studies have detected regions of persistent risk of natural transmission mainly along the Magdalena River valley, the region of Catatumbo, the Sierra Nevada de Santa Marta, the foothills of the eastern plains and Serrania de la Macarena. The departments with higher frequency of Chagas disease are thus: *Casanare, Santander, Norte de Santander, Cundinamarca, Boyacá, Meta, Arauca, Tolima, Huila and Bolivar* [13], [22]–[26].

A study showed that the costs of vector-control measures are low in comparison to that of the treatment for advanced forms of the disease [15]. Hence, the identification of population at risk of disease transmission is crucial to direct the primary prevention measures. As a consequence of several studies performed in the endemic countries, a better understanding of the biology of insect vectors, as well as its geographical distribution has been of great help in identifying regions where these insects are endemic. [27]. In line with this, some studies oriented to find infected vectors and to identify households at risk for Chagas disease, pointed out the state of *Casanare* in Colombia, as being the region with the highest risk of transmission of Chagas disease in the country in 2005 [26]. In the present study we aimed to assess the frequency of infection among people living in five municipalities of the state of *Casanare* in Colombia.

## Methods

### Ethics statement

This study was approved by the institutional board on research ethics at Universidad Antonio Nariño (Session No 080). Written informed consent was obtained from all participants (or from their parents in the case of minors) prior to answering the questionnaire and blood sampling. All participants received their confidential individual results of the test and those with positive results were advised to consult the local public health offices (and a notification of the number of infected patients detected was sent to the health surveillance office in each case).

### Design of the study

A cross-sectional study was conducted in which the target population was constituted by people aged 15–89 inhabiting the rural and urban areas of five municipalities in the department of *Casanare*, in Colombia. People were recruited by a public radio call for the diagnostic test of Chagas disease. Blood sampling was conducted in September and October, 2011. Serological tests were performed on a sample of individuals from the urban and rural populations of each municipality on spontaneous demand. The local urban hospitals and rural schools were the chosen locations for signing the written informed consent and blood sampling. All participants received their results in a closed envelope through the local health services in each community and medical advice on their respective infection status.

### Sample size

Sample size (n) was calculated with the following equations: x = Z (c/100)2r (100-r) and n = N x/((N-1) E2+x), where N is the population size, E is the margin of error (5%), r is the frequency of infection (50%), and Z (c/100) is the critical value for the confidence level c (5%). The estimated population is 44,355 for 2011 for the 5 selected municipalities and the age range of 15–89 according to Colombian demography government agency DANE (www.dane.gov.co). We initially assumed a margin of error of 5%, and an unknown prevalence.

The margin of error (E) was calculated with the following equations: *x = Z* (*^c^*/_100_)^2^ *r* (100-*r*) and *E = *Sqrt [^(*N*- *n*) *x*^/*_n_*_ (*N*-1)_], where *N* is the population size, *r* is the fraction of responses that we were interested in, and Z (c/100) is the critical value for the confidence level c.

### Study area

**Figure 1:**
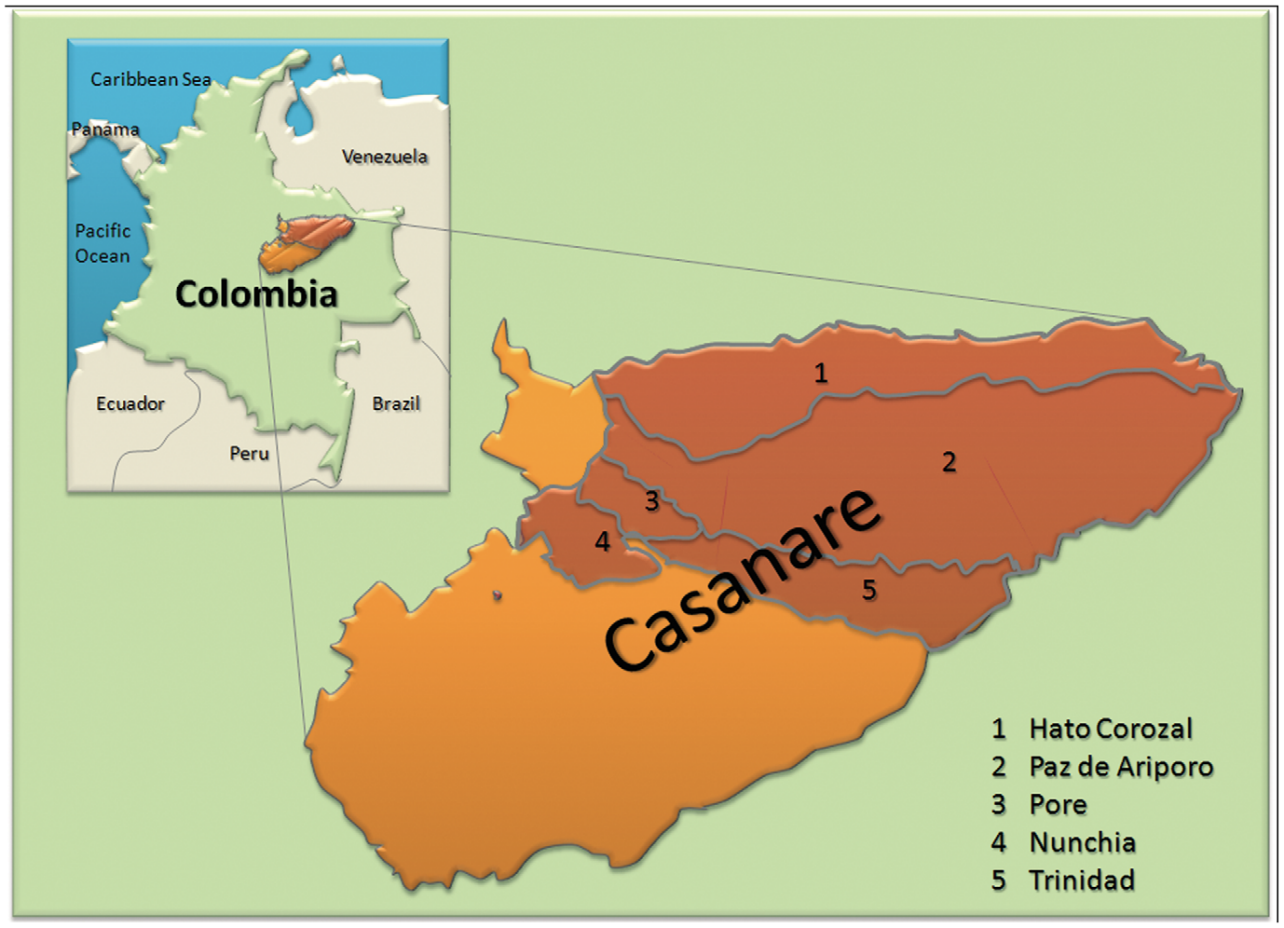
Map of the municipalities covered by this study.

The department of Casanare is located in the central eastern region in Colombia (5°21′0″N 72°24′36″W), with an extension of 44.490 km^2^ (**Figure 1**). The average height above sea level is 350 meters, with an average temperature of 26°C (ranging from 22 to 27°C). This department has the third highest human development index in Colombia, according to UN development program [28]. This study analyzed a sample of men and women between 15 and 89 years of age, in the rural and urban areas of the municipalities of *Hato Corozal, Nunchia, Paz de Ariporo, Pore and Trinidad* (**Figure 1**). As stated above, previous studies on the presence of infected vectors pointed out this department as at high risk for Chagas disease transmission.

### Data collection

A survey containing the following questions: birth date, marital status, place of residence, occupation, time living in the region, history of recent cardiovascular or digestive symptoms, personal or familiar history of Chagas disease or other previous diseases, history of being a blood donor/recipient, obstetric history, socioeconomic factors recognized as relevant for the risk of the infection like housing conditions, history of a relative with established diagnosis of Chagas disease, history of contact with the vector, ability to identify the vector, and previous tests for Chagas disease. The participants were interviewed by a trained physician.

### Enzyme-Linked ImmunoSorbent Assay (ELISA)

ELISA was performed with the commercial kit Chagatest ELISA recombinant, version 3.0 (Wiener Lab, Rosario, Argentina), which has a mixture of recombinant proteins from *T. cruzi* and has been widely used, with a demonstrated sensitivity of 98.81% (95%IC: 95.8–99.9) and a specificity of 99.62% (95%IC: 97.9–100) [29], [30]. Each serum was analyzed in duplicate, and the positive and negative controls were analyzed in triplicate. Optical density (OD) was measured at 450 and 650 nm. A sample was considered positive if the OD (subtracting OD650 from OD450) nm was ≥0.355 or negative if the OD was ≤0.354. Positive and negative controls used for specificity control, were always included to validate the results obtained.

### Hemagglutination

Hemagglutination assays were performed with the commercial kit Chagatest HAI (Wiener Lab, Rosario, Argentina), according to the manufacturer's instructions, the samples were treated with 2-mercaptoethanol (2-ME) at a dilution of 1∶40. This essay was read and interpreted manually. The samples were examined in five serial dilutions. A specimen was considered positive when agglutination occurred at 1∶24 dilution or more. Positive and negative controls used for specificity were always included to validate the results obtained. According to the manufacturer, experimental data from endemic and non-endemic populations and which were assayed by immunofluorescence and complement-fixation reaction (Machado & Guerreiro), demonstrated that in endemic populations, the specificity of this IHA method was 98% considering titers lower than 1/8 and its sensitivity was 95% considering titters higher or equal than 1/8 were confirmed by reference methods. In non-endemic populations, 100% of healthy individuals showed titers lower than 1/8 determined by IHA, 100% of individuals with positive serology confirmed by reference methods and parasitism confirmed by xenodiagnosis and/or hemoculture, higher or equal titers than 1/32 were observed determined by Chagatest IHA.

### Statistical analysis

Descriptive analyses were based on frequencies and percentages for qualitative variables, and means with their confidence intervals for quantitative variables. Bivariate analyses were performed using the Fisher's exact test to calculate odds ratios (OR) and the 95% confidence interval, using the version SPSS Statistics software (IBM). Data was analyzed using contingency tables for which participants were classified according to their infection status. Prevalence was calculated with the standard equation: Prevalence = (Number of positive persons/Population) *100.

## Results

**Figure 2:**
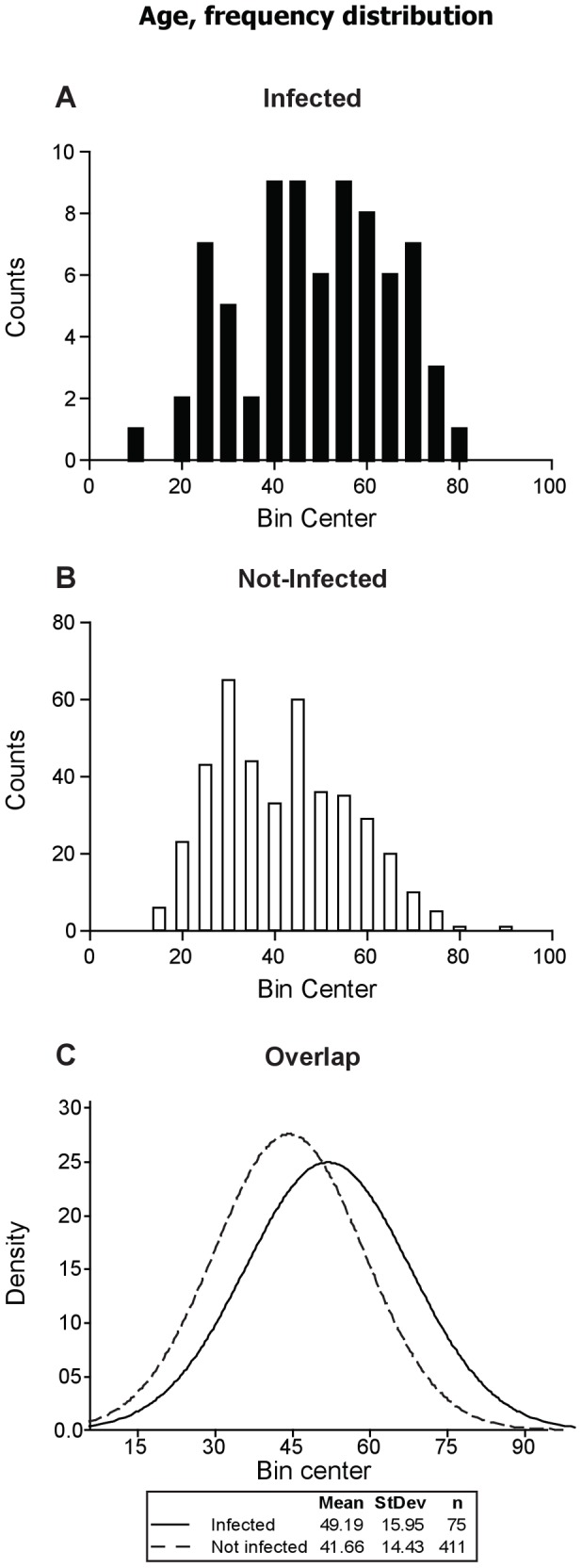
Distribution of frequency of age among infected and not infected participants. The frequency histograms of ages of participants from each group (infected, A; not infected, B. Overlapped distributions, C) is shown.

A map showing the geo localization of the surveyed communities is shown in **Figure 1**. The number of participants by gender and the total of infected individuals by sex and urban/rural place of living are shown in **Table 1**. A total of 486 people were sampled, ranging from 15 to 89 years of age. One 11 year old child was also included. The mean age among infected (49.19, SEM: 1.842) or not infected individuals (41.66, SEM: 0.7118) were different in the global sample (99% CI of differences: −12.28 to −2.784, p≤0.0001 in two-tailed unpaired t test) (**Figure 2** **.**). No differences were found analyzing the places of living (urban or rural domicile) or genders among infected or not infected participants.

**Table 1:** General characteristics of the study participants.

Characteristic	Number	%
**Total Sample**		
n	486	100%
Age, Mean (SEM)	42.82(0.67)	N.A.
Age, Median	42.74	N.A.
Rural	125	25.72%
Urban	361	74.28%
Male	203	41.77%
Female	284	58.44%
**Seropositive**		
N	75	15.43%
Age, Mean (SEM)	49.19 (1.84)	N.A.
Age, Median	49	N.A.
Rural	24%	0.05%
Urban	51%	0.10%
Male	26	5.35%
Female	49	10.08%
**Seronegative**		
N	411	84.57%
Age, Mean (SEM)	41.66(0.71)	N.A.
Age, Median	40.58	N.A.
Rural	102	24.81%
Urban	309	75.18%
Male	177	36.42%
Female	235	48.35%

SEM: Standard Error of the Mean.

The prevalence of serum antibodies against *T. cruzi* that we found in this study was 16.90% (**Table 2**). The frequencies of seropositive results by each municipality were as follows: Hato Corozal: 11.27%; Nunchia: 31.15%; Paz de Ariporo: 14.75%; Pore: 18.92%; Trinidad: 7.22%.

**Table 2:** Prevalence of seropositive results by gender and place of living.

*Total Sample*							
	Total	Female			Male		
	n	Total	Urban	Rural	Total	Urban	Rural
**Total**	486	284	219	65	202	141	61
%	100.00%	58.44%	45.06%	13.37%	41.56%	29.01%	12.55%
**Seropositive**	75	49	36	13	26	15	11
%	**15.43%**	**65.33%**	**48.00%**	**17.33%**	**34.67%**	**20.00%**	**14.67%**
**Seronegative**	411	235	183	52	176	126	50
%	84.57%	57.18%	44.53%	12.65%	42.82%	30.66%	12.17%

*Hato Corozal*							
	Total	Female			Male		
	n	Total	Urban	Rural	Total	Urban	Rural
**Total**	71	37	27	10	34	26	8
%	100.00%	52.11%	38.03%	14.08%	47.89%	36.62%	11.27%
**Seropositive**	8	5	3	2	3	2	1
%	**11.27%**	**62.50%**	**37.50%**	**25.00%**	**37.50%**	**25.00%**	**12.50%**
**Seronegative**	63	32	24	8	31	24	7
%	88.73%	50.79%	38.10%	12.70%	49.21%	38.10%	11.11%

*Nunchia*							
	Total	Female			Male		
	n	Total	Urban	Rural	Total	Urban	Rural
**Total**	61	37	30	7	24	15	9
%	100.00%	60.66%	49.18%	11.48%	39.34%	24.59%	14.75%
**Seropositive**	19	13	9	4	6	3	3
%	**31.15%**	**68.42%**	**47.37%**	**21.05%**	**31.58%**	**15.79%**	**15.79%**
**Seronegative**	42	24	21	3	18	12	6
%	68.85%	57.14%	50.00%	7.14%	42.86%	28.57%	14.29%

*Paz de Ariporo*							
	Total	Female			Male		
	n	Total	Urban	Rural	Total	Urban	Rural
**Total**	183	109	76	33	74	52	22
%	100.00%	59.56%	41.53%	18.03%	40.44%	28.42%	12.02%
**Seropositive**	27	18	12	6	9	5	4
%	**14.75%**	**66.67%**	**44.44%**	**22.22%**	**33.33%**	**18.52%**	**14.81%**
**Seronegative**	156	91	64	27	65	47	18
%	85.25%	58.33%	41.03%	17.31%	41.67%	30.13%	11.54%

*Pore*							
	Total	Female			Male		
	n	Total	Urban	Rural	Total	Urban	Rural
**Total**	74	50	43	7	24	16	8
%	100.00%	67.57%	58.11%	9.46%	32.43%	21.62%	10.81%
**Seropositive**	14	9	8	1	5	3	2
%	**18.92%**	**64.29%**	**57.14%**	**7.14%**	**35.71%**	**21.43%**	**14.29%**
**Seronegative**	60	41	35	6	19	13	6
%	81.08%	68.33%	58.33%	10.00%	31.67%	21.67%	10.00%

*Trinidad*							
	Total	Female			Male		
	n	Total	Urban	Rural	Total	Urban	Rural
**Total**	97	51	43	8	46	32	14
%	100.00%	52.58%	44.33%	8.25%	47.42%	32.99%	14.43%
**Seropositive**	7	4	4	0	3	2	1
%	**7.22%**	**57.14%**	**57.14%**	**0.00%**	**42.86%**	**28.57%**	**14.29%**
**Seronegative**	90	47	39	8	43	30	13
%	92.78%	52.22%	43.33%	8.89%	47.78%	33.33%	14.44%

Finally, we analyzed a number of variables among infected or not infected individuals (**Table 3**), and found that housing constituents like vegetable material, adobe or unfinished brick walls were associated with the infection (OR 1.777, 95% CI: 1.076 to 2.936, p = 0. 0317). Of note, 15% of infected individuals said they have donated blood, although no additional information was obtained on whether the donated blood was rejected or not transfused to other individuals. None of the infected patients received a blood transfusion. The fact of having previously screened for Chagas disease was also associated with the infection (OR 4.030, 95% IC: 2.211 to 7.346, p<0.0001), as was the case in 7 individuals which already know that they were infected. Having a relative diagnosed with Chagas disease has been previously described as a risk factor for Chagas disease, but in our study we did not find it as a factor associated with the infection.

**Table 3:** Contingency analyses for the studied factors potentially associated with Chagas disease.

	Seropositive		Seronegative		OR	95% CI	P value
	n	*%*	n	%			
**Housing materials: vegetal material, adobe or unfinished brick walls.**	33/75	44.00	126/411	30.00	1.777	1.076 to 2.936	**0.0317**
**Have ever donate blood**	11/70	15.71%	95/406	23.40%	0.6103	0.3081 to 1.209	0.1652
**Have ever received a blood transfusion**	0/75	0.00%	10/405	2.47%	0.2494	0.01445 to 4.306	0.3743
**Previous Test for Chagas disease**	22/74	29.73%	38/400	9.50%	4.030	2.211 to 7.346	**<0.0001**
**Previously diagnosed with Chagas**	7/74	9.46%	5/399	1.25%	8.233	2.538 to 26.71	**0.0007**
**Relative diagnosed with Chagas**	15/71	21.13%	63/401	15.71%	1.437	0.7652 to 2.699	0.2972
**Contact with Blood of Wild animals**	38/69	55.07%	161/335	48.06%	1.325	0.7871 to 2.230	0.2941
**Able to identify the insect vector**	54/73	73.97%	302/391	77.24%	0.8376	0.4718 to 1.487	0.5481
**Have ever seen insects vectors in household the last 5 years**	32/73	43.84%	156/391	39.90%	0.9452	0.5812 to 1.537	0.9018
**Believe that have been ever bitten by insect vectors**	28/59	47.46%	129/219	58.90%	0.6302	0.3536 to 1.123	0.1391
**Women with history of abortion at any gestational age**	5/49	10.20%	18/235	7.66%	0.8342	0.2825 to 2.464	1
**Women with children under 10 years of age**	14	N/A	71	N/A	N/A	N/A	N/A
**Symptoms (excluding those patients with history of any cardiovascular disease)**							
**Cough (mainly at night)**	4/56	7.14%	10/274	3.65%	2031	0.6133 to 6.725	0.2689
**Palpitations**	3/56	5.36%	7/274	2.55%	2159	0.5407 to 8.621	0.3826
**Lipotimia**	3/56	5.36%	12/274	4.38%	1236	0.3370 to 4.532	0.7262
**Precordial pain**	22/56	39.29%	73/274	26.64%	1782	0.9782 to 3.245	0.074
**Dyspnea**	8/56	14.29%	30/274	10.95%	1356	0.5856 to 3.138	0.4916
**Two or more of the above symptoms**	10/56	17.86%	28/274	10.22%	1910	0.8686 to 4.199	0.1104
**Dysphagia**	7/56	12.50%	16/274	5.84%	2304	0.9003 to 5.894	0.0855
**No symptoms**	39/56	69.64%	175/274	63.87%	1298	0.6976 to 2.415	0.4460

## Discussion

In this investigation we aimed at appraise the frequency of the infection among adults in five municipalities in the north of the department. These municipalities sum a total area of 24,517 km^2^ (55.10% of the department's area) and concentrate 24.22% of the population between 15 and 89 years old in the department, according to estimations by the National Department of Statistics (DANE). To our knowledge, this is the first study on the prevalence of the infection by *T. cruzi* among adult females and males residing these municipalities in *Casanare*. However, a large-scale study involving all municipalities in the region (comprising those from the neighbor departments of Arauca and Boyacá) is currently required to identify the magnitude of the human infection and to evaluate the effects of the interventions performed to control of *T. cruzi* infection (in addition to the vector-oriented studies).

We have selected these municipalities considering data from a survey performed in children of 7 municipalities in 2004, which found that the municipality with the highest prevalence of the infection was Hato Corozal, followed by Nunchia and Paz de Ariporo. Moreover, the data from the blood bank of Yopal was also considered in this study [31]. However, the prevalence among adults remained to be studied. Trinidad was selected as it is farther from the mountains, and no information was found about prior prevalence in this municipality. Two previous studies on serology for *T. cruzi* in blood donors in banks around Colombia, found that the department with the highest proportion of seropositive results is *Casanare*, with 12.6% (111/881) [15] and 7.2% (107/1487) [20], respectively. As we cannot assume that the sample analyzed in these two studies (conducted in *Yopal*, the largest city of Casanare) is representative of the population of the entire region, we calculated the sample size in two scenarios: the one where the prevalence would be about 7.5% (based on these previous studies) and another with unknown prevalence (in this case we use 50% in the equation). This allowed to estimate that a sample of 107 individuals in the first scenario or 381 individuals in the second scenario will be sufficient to find the prevalence [32], [33] with a margin of error of 5% and a confidence of 95%.

Herein we found a prevalence of *T. cruzi* infection of 16.91%, in people from 5 of the 19 municipalities of the state of *Casanare* in Colombia. This prevalence is high and is in accordance with data from the national study of seroprevalence and risk factors for Chagas disease, conducted in 1999, which found a prevalence of infection of 35 per 1,000 for children less than 15 years, mainly in the eastern region. In studies of morbidity in adults, seropositivity between 19.4% and 47% has been reported. Later, a study involving the analysis of infected vectors and their presence in housings that showed this region as the one with the highest risk of disease transmission in Colombia [26]. More recently, a study found a prevalence of 4% of infection among pregnant women and 9.3% among their relatives in the same population [12]. Hence, it is of great importance to implement well-organized surveillance strategies in this population, in order to the prompt detection of infected people, and most importantly, the implementation of a follow up program for these patients.

In this study we did not measure the stage of the disease among the infected individuals. However, as this investigation was performed in the rural and urban locations, obtaining a chest x-ray and electrocardiogram/echocardiogram from the participants was not possible. This is a relevant limitation of our study, and we have remitted the infected patients to their health insurance services in order to address clinical classification in each case. But it is possible to speculate that most patients are in the indeterminate form of the disease. The recommended measures in these patients include a close clinical follow-up, to assess the extent of cardiac commitment by the disease [34]. Therapeutic options are benznidazole and nifurtimox which has been developed more than three decades ago. These medication exhibit a high toxicity and their effect on the prevention of disease progression remains controversial [9]. But, based on results from randomized and observational studies in children, the etiological treatment is indicated in individuals on the indeterminate phase of the disease, and should be prescribed by a physician experienced in the management of these medications and being able to diagnose and deal with possible side effects, and ensure follow-up after treatment [34]. Definitive evidence on this subject will come from the BENEFIT trial, an ongoing international multicentric, randomized, double-masked, placebo-controlled investigation that is evaluating the clinical outcome after 6 years of follow-up in patients with chronic Chagas disease cardiomyopathy treated with benznidazole [35].

Of note, the average age among infected patients was higher than that of not infected participants (**Table 1**). This may be related to the impact of previously implemented primary measures of control of transmission in this population, and is in agreement with the observation of an age >29 years as a risk factor for the infection in pregnant women in a recently published study in the same population [12].

The Department of Casanare occupies about 4% of the territory of Colombia, with an extension of 44,640 km^2^ and is located northwest of the Colombian Orinoco geographical region. Bordered on the north by the department of Arauca by the Casanare River, to the east by the department of Vichada by Meta River, on the south by the department of Meta through Upía and Meta rivers and west by the departments of Boyacá and Cundinamarca. Due to influence of altitude, the temperature ranges between 27°C in the lowlands and 6°C on the elevated areas. About 95% of the territory has a warm humid climate of the foothills of the mountains and the plains, while the remaining 5% exhibit four different climates: humid medium with 3.6% of the Department, cold and very wet 1% extremely cold and rain and 0.03%. Although the climate behavior is relatively uniform throughout the year, February and March are the months with higher temperatures and June and July the coldest. The relative humidity varies between 60 and 90%. The five municipalities included in this study exhibit a warm humid climate. Politically, the Department consists of 19 municipalities: Aguazul, Chameza, Hato Corozal, La Salina, Mani, Monterrey, Nunchia, Orocué, Paz de Ariporo, Pore, Recetor, Sabanalarga, Sacama, San Luis de Palenque, Tamara, Tauramena, Trinidad, Villanueva and Yopal.

The territory of Casanare comprises a diverse ecosystem, due to its high altitudinal variation. Casanare contains approximately 3,300 km2 of the Cordillera Oriental and represents 12.83% of the Orinoco River basin in Colombia. It has a complex ecosystem with combination of mountain slopes (10%), Piedmont (20%) and sheets (70%), where biodiversity has historically shared the land with livestock grazing by the traditional prairie. These conditions have contributed to the maintaining of the sylvatic cycle of *T. cruzi* infection, as several mammal species may act as hosts for the parasite. In line with this, the presence of native palm tree (*Attalea butyracea*) near the housings has been recognized as a risk factor for Chagas disease in some municipalities in Casanare and Arauca [27], [36]. Indeed, an intense colonization by wild *Rhodnius prolixus* and other *Triatominae* species was found not only in *A. butyracea*, but also in agro-industrial plantations of *Elaeis guineensis*, that are being used as a renewable source of vegetable oil to generate biodiesel. The frequencies of colonization were 92.8 and 100% respectively. Further analysis showed that 67 and 41% of these insects were naturally infected with *T. cruzi*, respectively [27]. Although no industrial plantations of *E. guineensis* are found in the areas included in the present study (some large plantations of rice and mostly low-scale maize and banana plantations are found instead), the native *A. butyracea* is commonly found in these municipalities.

Among the surveyed municipalities, we found a seroprevalence ranging from 7.22% to 31.15%. The higher seroprevalence was observed in *Nunchia*, confirming the data from previous studies on the presence of infected vectors. Conversely, Trinidad was the municipality with the lowest seroprevalence. It is possible that among the municipalities included in the present study, differences in the presence of *A. butyracea* near the houses may play a role in determining different prevalences of *T. cruzi* infection in adult humans. Alternatively, the above mentioned differences in the seroprevalence may be in association with a higher frequency of artisan materials in the housings in Nunchia. In fact, in this municipality, 68.42% of the infected people were living in houses considered of risk for disease transmission (i.e., housing materials like vegetable material, adobe or unfinished brick walls), while these materials were present in the houses of 35.09% of infected people from all the other municipalities. Housing materials like these have been largely reported as risk factors for the infection, as we show in **Table 3** for the sample of this study (OR 1.77, p = 0.0317 95%IC 1.076–2.936). Other social and political factor may however have concurred to favor such high prevalence of infection. The rural areas of this municipality have been until recently, characterized by the presence of illegal armed groups, which may have hindered the development of vector control activities.

The study by Cucunuba *et. al.* [12] was focused on measuring the prevalence of *T. cruzi* infection in pregnant women seeking health care at the health centers of Yopal. They included a total of 982 pregnant women, aged 13–46 years, and found a global prevalence of *T. cruzi* infection of 3.97%. Of note, women that were from Hato Corozal and Nunchia but went to Yopal in search of medical assistance, showed increased prevalences of the infection (20% and 13% respectively). Differently, our study did not include any pregnant women and sampling was conducted *in loco* in the respective municipalities. These differences in the results from the two studies are probably due to differences in the target population as well as the location of sampling. Indeed, the analysis of relatives of infected women showed a higher prevalence of 9.3%. Additional risk factors that were found by previous studies include low education level and rural residency [12]. However, in our study we did not find significant differences in the educational level among infected and not infected people nor in the place of residence (data not showed).

We found that those individuals who had a previous positive screening test for Chagas disease were more prone to obtain a positive result in our study (OR 4.03, p<0.0001 95%IC 2.211–7.346), as is the case for seven individuals who have an established diagnosis of infection. None of these patients had received specific treatment for Chagas disease. Patients with previous screening for Chagas disease and inconclusive or negative results may be more motivated to participate in our study, since they may want to confirm or discard their infection. That is in association with the fact that having a previous blood test for Chagas disease was more common among the individuals that were positive in our study.

The main limitation of this study is the fact that the sample was not randomized, essentially due to the characteristics of the distribution of the population in a vast territory (0.01 inhabitant/km^2^). Randomizing is not possible outside of the urban centers. To attempt to solve this problem we used various strategies: first, a public call was made on the radio at least 4 weeks before the blood sampling journeys, inviting people to receive a free diagnostic test for Chagas disease. Second, the blood sampling points were settled on Sundays, where most people are out of their works and able to participate. Third, in the case of rural regions, a mobile station was used for the blood sampling house by house.

According to WHO, the diagnosis of Chagas disease in endemic countries requires two positive results in serology tests [6], [37]. In the present study we first analyzed all samples with an ELISA kit which uses a mixture of recombinant proteins from *T. cruzi*, and has been widely used with a good performance in terms of sensitivity and specificity [30]. All the ELISA positive cases (and 3 negative cases) were subsequently analyzed by Immuno hemagglutination assay using other commercially available kit. The concordance of results between positive ELISA and positive IHA was 100% as previously reported [30]. Hence, seropositive individual found in the present study are confirmed cases of infection with *T. cruzi*.

In addition to the serologic tests, quantitative real-time PCR is a highly specific technique that is being proposed for the diagnosis of *T. cruzi* infection, but its lack of sensitivity in the clinical context, makes it a not suitable diagnostic tool for Chagas disease at the moment [38]. Following the governmental directive for the diagnosis and treatment of Chagas disease, blood samples from all these patients should be referred by their medical insurance providers to the national institute of health in Colombia, in order to be reported and monitored [39].

Our results underline the need for sanitary authorities to reinforce their activities directed to the detection of infected people as well as to the control of Chagas disease transmission in this region. Whereas vector control measures have been implemented by the local govern and have been regularly carried out in all the municipalities of Casanare since 1996, their coverage has been partial (i.e. 53.3% of high risk housings were sprayed with insecticides), mostly because some rural areas overlap with areas of presence of illegal armed groups, which hinders the sustainability of surveillance and control measures. Moreover, the populations of insects living outside of human housings represent a major challenge in the vector control strategies, since they can be a source of re-infestation of houses that have been already intervened with insecticides [27]. The present study provides evidence for a considerable number of infected people that, although are mostly asymptomatic at the moment of the study, may eventually develop Chagas disease cardiomyopathy and thus are suitable for secondary prevention measures. Finally, we hope our data will call the attention of sanitary authorities, to implement efficient measures oriented to the identification of infected adults and their follow-up. Considering that the region has been at high risk for the transmission of this infection, this is of the most relevance.

## Supporting Information

Checklist S1 Strobe statement - checklist for reporting observational studies.
(DOC)
