# Supplementary material for: Prevalence of Trypanosoma cruzi Infection among People Aged 15 to 89 Years Inhabiting the Department of Casanare (Colombia)
Source: PLoS Negl Trop Dis. 2013 Mar 7;7(3):e2113. doi: 10.1371/journal.pntd.0002113 (PMC3591340; doi:10.1371/journal.pntd.0002113)
Supplement: Checklist S1 — Strobe statement - checklist for reporting observational studies. (DOC) [file pntd.0002113.s001.doc]

STROBE Statement—Checklist of items that should be included in reports of ***observational studies***

|  | Item No | Recommendation |
| --- | --- | --- |
| **Title and abstract** | 1 | (*a*) Indicate the study’s design with a commonly used term in the title or the abstract  The tile is: Prevalence of *Trypanosoma cruzi* infection among people aged 15 to 89 years inhabiting the Department of *Casanare* (Colombia). |
|  |  | (*b*) Provide in the abstract an informative and balanced summary of what was done and what was found.  Lines 3-7 and 11-16 of the abstract. |
| Introduction | | |
| Background/rationale | 2 | Explain the scientific background and rationale for the investigation being reported  Paragraph 7. |
| Objectives | 3 | State specific objectives, including any prespecified hypotheses  First line of the abstract. |
| Methods | | |
| Study design | 4 | Present key elements of study design early in the paper  The study design is declared in the abstract (3^rd^ line)  Design of the study is also a section of the methods. |
| Setting | 5 | Describe the setting, locations, and relevant dates, including periods of recruitment, exposure, follow-up, and data collection.  The study area section of methods includes the geographical information. Data collection section of methods describes how the data was collected. |
| Participants | 6 | (*a*) Give the eligibility criteria, and the sources and methods of selection of participants  These informations are included in the Design of the study section of the methods |
| Variables | 7 | Clearly define all outcomes, exposures, predictors, potential confounders, and effect modifiers. Give diagnostic criteria, if applicable  Methods section (see ELISA and HAI), and paragraph 14 of Discussion. |
| Data sources/ measurement | 8* | For each variable of interest, give sources of data and details of methods of assessment (measurement). Describe comparability of assessment methods if there is more than one group.  Provided in the Statistical analysis section of Methods and in Tables 1-3. |
| Bias | 9 | Describe any efforts to address potential sources of bias  Paragraph 11 of discussion. |
| Study size | 10 | Explain how the study size was arrived at  Sample size section of Methods. |
| Quantitative variables | 11 | Explain how quantitative variables were handled in the analyses. If applicable, describe which groupings were chosen and why  Provided in the Statistical analysis section of Methods |
| Statistical methods | 12 | (*a*) Describe all statistical methods, including those used to control for confounding  Statistical analysis section of Methods |
|  |  | (*b*) Describe any methods used to examine subgroups and interactions  Statistical analysis section of Methods |
|  |  | (*c*) Explain how missing data were addressed  N/A |
|  |  | (*d*) If applicable, describe analytical methods taking account of sampling strategy  Sample size section of Methods |
|  |  | (*e*) Describe any sensitivity analyses  N/A |
| Results | | |
| Participants | 13* | (a) Report numbers of individuals at each stage of study—eg numbers potentially eligible, examined for eligibility, confirmed eligible, included in the study, completing follow-up, and analysed  First paragraph of methods. |
|  |  | (b) Give reasons for non-participation at each stage  N/A |
|  |  | (c) Consider use of a flow diagram  N/A |
| Descriptive data | 14* | (a) Give characteristics of study participants (eg demographic, clinical, social) and information on exposures and potential confounders  Provided in table 1. |
|  |  | (b) Indicate number of participants with missing data for each variable of interest  Tables1-3. |
| Outcome data | 15* | Report numbers of outcome events or summary measures  N/A |
| Main results | 16 | (*a*) Give unadjusted estimates and, if applicable, confounder-adjusted estimates and their precision (eg, 95% confidence interval). Make clear which confounders were adjusted for and why they were included  Table 3. |
|  |  | (*b*) Report category boundaries when continuous variables were categorized  N/A |
|  |  | (*c*) If relevant, consider translating estimates of relative risk into absolute risk for a meaningful time period  N/A |
| Other analyses | 17 | Report other analyses done—eg analyses of subgroups and interactions, and sensitivity analyses  N/A |
| Discussion | | |
| Key results | 18 | Summarise key results with reference to study objectives  In this investigation we aimed at appraise the frequency of the infection among adults in five municipalities in the north of the department.  Herein we found a prevalence of *T. cruzi* infection of 16.91%, in people from 5 of the 19 municipalities of the state of *Casanare* in Colombia. This prevalence is high and is in accordance with data from the national study of seroprevalence and risk factors for Chagas disease, conducted in 1999, which found a prevalence of infection of 35 per 1,000 for children under 15 years, mainly in the eastern region. In studies of morbidity in adults it was found a seropositivity between 19.4% and 47%. In this study we did not measure the stage of the disease among the infected individuals. However, as this investigation was performed in the rural and urban locations, obtaining a chest x-ray and electrocardiogram/ echocardiogram from the participants was not possible. This is a relevant limitation of our study, and we have remitted the infected patients to their health insurance services in order to address clinical classification in each case. But it is possible to speculate that most patients are in the indeterminate form of the disease. |
| Limitations | 19 | Discuss limitations of the study, taking into account sources of potential bias or imprecision. Discuss both direction and magnitude of any potential bias  The main limitation of this study is the fact that the sample was not randomized, essentially due to the characteristics of the distribution of the population in a vast territory (0,01 inhabitant/km²). Randomizing is not possible outside of the urban centers. To attempt to solve this problem we used various strategies: first, a public call was made on the radio at least 4 weeks before the blood sampling journeys, inviting people to receive a free diagnostic test for Chagas disease. Second, the blood sampling points were settled on Sundays, where most people are out of their works and able to participate. Third, in the case of rural regions, a mobile station was used for the blood sampling house by house. |
| Interpretation | 20 | Give a cautious overall interpretation of results considering objectives, limitations, multiplicity of analyses, results from similar studies, and other relevant evidence.  *T. cruzi* infection is highly prevalent in the north region of *Casanare*, in Colombia. |
| Generalisability | 21 | Discuss the generalisability (external validity) of the study results  Our results underline the need for sanitary authorities to reinforce their activities directed to the detection of infected people as well as to the control of Chagas disease transmission in this region. Whereas vector control measures have been implemented by the local govern and have been regularly carried out in all the municipalities of Casanare since 1996, their coverage has been partial (i.e. 53.3% of high risk housings were sprayed with insecticides), mostly because some rural areas overlap with areas of presence of illegal armed groups, which hinders the sustainability of surveillance and control measures. Moreover, the populations of insects living outside of human housings represent a major challenge in the vector control strategies, since they can be a source of re-infestation of houses that have been already intervened with insecticides [29]. The present study provides evidence for a considerable number of infected people that, although are mostly asymptomatic at the moment of the study, may eventually develop Chagas disease cardiomyopathy and thus are suitable for secondary prevention measures. Finally, we hope our data will call the attention of sanitary authorities, to implement efficient measures oriented to the identification of infected adults and their follow-up. Considering that the region has been at high risk for the transmission of this infection, this is of the most relevance. |
| Other information | | |
| Funding | 22 | Give the source of funding and the role of the funders for the present study and, if applicable, for the original study on which the present article is based  This study was entirely financed by Universidad Antonio Nariño, under the project number: 435 2010229. The funders had no role in study design, data collection and analysis, decision to publish, or preparation of the manuscript. |
